# Extraction of user's navigation commands from upper body force interaction in walker assisted gait

**DOI:** 10.1186/1475-925X-9-37

**Published:** 2010-08-05

**Authors:** Anselmo Frizera Neto, Juan A Gallego, Eduardo Rocon, José L Pons, Ramón Ceres

**Affiliations:** 1 Bioengineering Group, Consejo Superior de Investigaciones Cientificasi, Crta. Campo Real Km 0.200, Arganda del Rey - Madrid, Spain

## Abstract

**Background:**

The advances in technology make possible the incorporation of sensors and actuators in rollators, building safer robots and extending the use of walkers to a more diverse population. This paper presents a new method for the extraction of navigation related components from upper-body force interaction data in walker assisted gait. A filtering architecture is designed to cancel: (i) the high-frequency noise caused by vibrations on the walker's structure due to irregularities on the terrain or walker's wheels and (ii) the cadence related force components caused by user's trunk oscillations during gait. As a result, a third component related to user's navigation commands is distinguished.

**Results:**

For the cancelation of high-frequency noise, a Benedict-Bordner g-h filter was designed presenting very low values for Kinematic Tracking Error ((2.035 ± 0.358)·10^-2 ^*kgf*) and delay ((1.897 ± 0.3697)·10^1^*ms*). A *Fourier Linear Combiner *filtering architecture was implemented for the adaptive attenuation of about 80% of the cadence related components' energy from force data. This was done without compromising the information contained in the frequencies close to such notch filters.

**Conclusions:**

The presented methodology offers an effective cancelation of the undesired components from force data, allowing the system to extract in real-time voluntary user's navigation commands. Based on this real-time identification of voluntary user's commands, a classical approach to the control architecture of the robotic walker is being developed, in order to obtain stable and safe user assisted locomotion.

## Background

Walkers are designed to assist pathological gait, helping in balance, and providing weight support to the user. Moreover, as walkers rely on the user's ability to walk, these devices play an important role in empowering user's rehabilitation. Walker-assisted gait is a theme of interest in the scientific community. Studies regarding walker assisted gait are found in [1-3]. Conventional walkers are prescribed according to certain user's characteristics:

1. *Standard or four-legged wakers *are useful for patients with poor balance, [4], or for those that require some level of partial body weight support (PBWS), at the cost of compromising gait patterns and posture during gait. Upper body strength and good motor coordination are demanded for lifting up and placing forward the device during gait, [5].

2. *Rollators or four-wheeled walkers *offer more natural gait patterns but lack in stability. If users should put much weight on the device, it may roll away, resulting in a fall. In that context, rollators should be used by patients that require minimal weight bearing, such as individuals with mild to moderate Parkinson's disease or ataxia, [5].

The advances in technology make possible the incorporation of sensors and actuators in such devices, bringing to these devices new characteristics: improved the therapies based on walkers by means of assist-as-needed intervention and improved device's reliability. These new characteristics extended the use of walkers to a more diverse population. (Robotic, advanced or smart)-walkers are normally three/four-wheeled devices in which locomotion is controlled by motors, offering, at the same time, natural gait patterns, lateral stability and the possibility of PBWS. Sensors aimed at extracting user or environment conditions provide safe and efficient control. Some examples of the most significant smart walkers in the literature are found in [6-11]. A review regarding such devices along with a functional classification was presented in [12].

In the framework of Simbiosis Project, a robotic walker equipped with a multimodal user-machine interface was developed, [12]. This work presents a new method for the extraction of user's navigation commands from upper-body force interaction in walker assisted gait. After a previous analysis of the force sensor data measured in walker's handles, the main components were identified. First, a high-frequency component originated from the vibrations introduced by the wheels/floor irregularities was found. These components can be attenuated by improving the device's structure. Nevertheless, in outdoors the pavement usually presents imperfections. This requires the development of efficient techniques to remove these components from force data.

Second, a component related to user's trunk oscillations, and consequently to user's gait, is observed. In previous works, [13], such component was characterized and continuously monitored in order to infer gait parameters from force data. Nevertheless, in this work the focus is on the third component related to user's navigation commands. It is fundamental to infer such commands from the interaction with the robotic walker for an efficient control of the device during assisted gait.

This paper presents a filtering architecture and its validation for obtaining user's navigation commands. Section 2 includes a brief presentation of the Simbiosis walker, the force measurement configuration and, most importantly, a discussion regarding the filtering designed to extract the components related to user's navigation commands. In section 3, the experimental results are presented along with the corresponding discussion. Finally, section 4 presents the conclusions and future work.

## Methods

**Figure 1:**
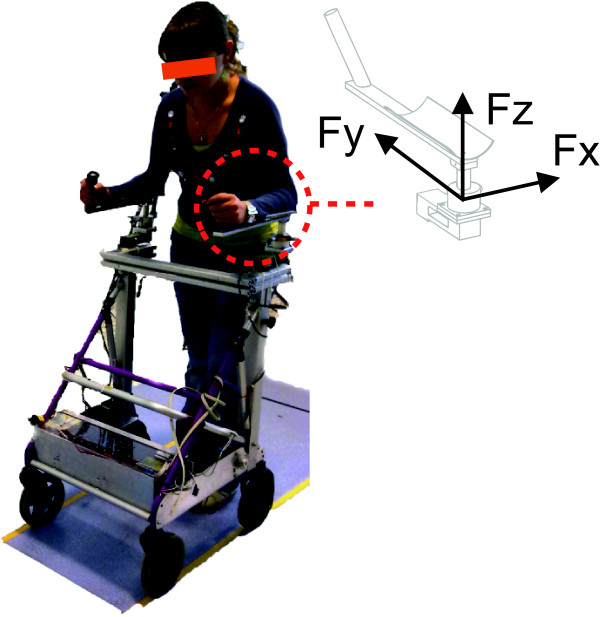
**Illustration of the SIMBIOSIS walker upper body force interaction subsystem**.

The robotic walker developed under the framework of the Simbiosis Project presents a series of sensor subsystems designed for the acquisition of gait parameters and for the characterization of the human-robot interaction during gait, [12]. One of them, the *upper-body force interaction subsystem*, is based on two tridimensional (3D) force sensors installed under the forearm supporting platforms, Figure 1. Each 3D force sensor is compound by one MBA400-200Lb biaxial sensor by Futek and one Transdutec TPP-3/75 load cell with their respective amplifiers. The biaxial sensors are used for measuring the *X *(lateral direction) and *Y *(advance direction) components. The load cells measure the *Z *component (vertical direction). Force sensors are integrated into a real-time architecture based on Matlab Real-Time xPC Target Toolbox. When data storage for offline studies is required, a laptop computer is also introduced into the system's architecture. The laptop PC also adds the possibility to control the system externally through a wireless LAN remote desktop connection.

**Figure 2:**
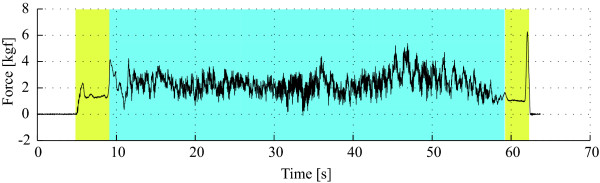
**Typical raw force data obtained from the Y-axis of the 3D sensor**. Two main zones highlighted: (i) the yellow zone indicates the moments in which the subject has his arms resting on the structure of the walker and is not walking, and (ii) denotes the moments in which the subject is actually walking with the device. Three main components are identified in force signals: the vibrations introduced by floor/walker wheels imperfections, oscillations due to user's trunk motion during gait and the voluntary transient events related to the user's navigational commands.

A previous study regarding the forces acquired during experiments of assisted gait lead to the identification of three main components in force signals: the vibrations introduced by floor/walker wheels imperfections, oscillations due to user's trunk motion during gait and the voluntary components related to the user's navigational commands. The typical force data acquired on the *y *axis of one of the force sensors is presented in Figure 2. As it can be seen, in the instants that the subject is not walking but has his arms resting on the forearm supports (yellow area in Figure 2), no high frequency noise is observed. This indicates that the high frequency components are generated during the movement of the device. As observed by the authors, such noise is caused by vibrations introduced by both irregularities on the ground and imperfections on the surface of the walker's wheels.

In addition, during the moments in which the subject is walking (blue highlighted area), slower oscillations are also observed in all axis of force data. In previous works, the authors demonstrated that this oscillatory component is specially observed in the vertical direction of the force data, and is a result of the lateral displacements of user's trunk, [14]. Such oscillations are translated into forearm reactions as the user is supported by the walker. Movements of user's trunk and, consequently, user's center of gravity (CoG) are highly correlated with gait phases, [15]. In [13], the authors proposed a methodology for the extraction of gait parameters, such as heel-strike, toe-off and cadence, from this force component.

Finally, transient events related to user's navigation commands are also found within the force sensor data. The main objective for the installation of force sensors in walker's handles was the identification and characterization of user's guidance intentions. As an example, at the beginning of the blue area in Figure 2, a high amplitude peak identifying the initial pushing force to move the device is observed. More information related to guidance commands is within this force signal, but they can not be easily identified without its proper extraction from the two previously commented components. Next section introduces the new methodology for the extraction of the components related to user's guidance intentions.

For the validation of the filtering methodology following presented, five healthy subjects were asked to walk with the device in a 40 m track prepared for the experiments. The track was placed in a indoors installation and included a 90 degrees curve at the center. The five subjects were asked to walk, at preferred speed, three times in each direction, resulting in a total of 30 repetitions. It took from 50 to 70 seconds for the subjects to complete the track each time. During the experiments, force data was acquired at 1 KHz and stored for the analysis presented in the following sections. Informed consent was obtained from the patients that participated in this study.

The subjects recruited for the proposed experiments presented no history of any dysfunction on either upper or lower limbs. At this point in the study, reference signals and validation of the method are the main objectives. Individuals with pathological gait will be addressed in the future.

### Data analysis

**Figure 3:**
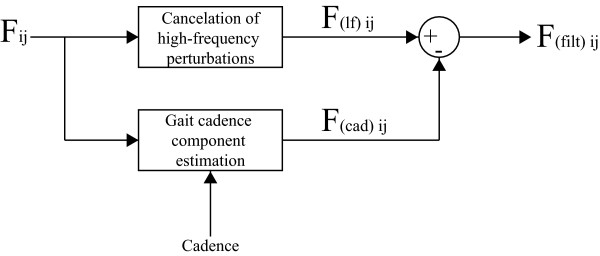
**Signal processing architecture for user's intent component separation**. *F*_*ij *_is the force signal obtained from axis (j), sensor (i). $F_{{\left(\iota f\right)}_{ij}}$ denotes the force signal obtained after the high frequency noise cancelation and $F_{{\left(cad\right)}_{ij}}$ is the periodical, cadence-dependent component of force. Finally, $F_{{\left(filt\right)}_{ij}}$ is the processed final signal related to user's navigation intent.

Considering the obtained knowledge regarding the several components contained in the force signals, the authors propose the filtering strategy presented in Figure 3. On the one hand, the upper branch is designed for the cancelation of high-frequency components that result from vibrations caused by wheels/floor irregularities.

On the other, the lower branch is constructed to online estimate the component caused by user's trunk oscillations and, therefore, highly correlated with user's cadence. This last component is, then, subtracted from the force data filtered by the first block. As it can be seen, in addition to the force signals, cadence is also an input for this filter. The idea is to selectively and adaptively filter the force data without compromising the amplitude of components which frequencies are close to gait cadence as they can contain relevant information regarding user's intents.

#### Design of high-frequency noise cancelation filter

The technique presented in this section relies on the high-frequency of the force components related to the vibrations of the walker's structure. Classical low-pass filters, such as Butterworth, Chebyshev, among others, can be used for the cancelation of high-frequency components of the acquired force signals, nevertheless, such approach would also introduce an important phase shift between input and outputs signals causing a temporal delay on the filtered signal. Such situation is undesirable in real-time applications once delay affect the cognitive interaction between the walker and the user.

In this context, for this first stage of noise cancellation an approach based on *g-h filters *is designed. G-h filters are simple recursive filters that estimate future position and velocity of a variable based on first order model of the process. Measurements are used to correct these predictions, minimizing the estimation error. Traditional applications of g-h filters are radar tracking and aeronautics, [16]. The general form of a g-h filter is described in the following equations.

(1) $$x_{k,k}=x_{k,k-1}+g_{k}(y_{k}-x_{k,k-1})$$

(2) $${\dot{x}}_{k,k}={\dot{x}}_{k,k-1}+\frac{h_{k}}{T_{s}}(y_{k}-x_{k,k-1})$$

(3) $${\dot{x}}_{k,k}={\dot{x}}_{k,k-1}+\frac{h_{k}}{T_{s}}(y_{k}-x_{k,k-1})$$

(4) $$x_{k+1,\;k}=x_{k,k}+T_{s}{\dot{x}}_{k,k}$$

Equations 1 and 2 are designated as update, tracking, or filtering equations. They estimate the current position, *x*_*k, k*_, and velocity, ${\dot{x}}_{k,k}$, of the variable based on previous predicted position, *x*_*k, k*-1_, and velocity, ${\dot{x}}_{k,k-1}$, taking the current measurement *y*_*k *_to account. Confidence on measures is weighted by gains *g*_*k *_and *h*_*k*_. Equations 3 and 4 are called prediction equations as they provide a prediction of future position and velocity, *x*_*k*+1,*k*_, ${\dot{x}}_{k+1,k}$, based on first order dynamic model of the variable. As g-h trackers consider a constant velocity model, predicted velocity ${\dot{x}}_{k+1,k}$ is equal to the current one, ${\dot{x}}_{k,k}$. The assumption of constant speed is reasonable considering that human movements are slow, presenting small accelerations, [17], and that the data is sampled at high rates (in this study, *f*_*sampling *_= 1*kHz*).

G-h filters are affected by two error sources, [16]: (i) the lag, dynamic, bias or systematic error, which are related to the constant velocity assumption, and (ii) the measurement error, which is inherent to the sensor and measurement process. Typically, the smaller *g_k _*and *h_k _*are, the larger is the dynamic error and the smaller are the measurement errors, [16]. In designing a g-h tracking filter there is a degree of freedom in choice of the relative magnitude of the measurement and dynamic errors.

To simplify the selection of filter gains (*g_k_, h_k_*), two filters that are optimal in some sense are considered. These filters are the Benedict-Bordner Filter (BBF) and the Critically Dampened Filter (CDF). BBF minimizes the total transient error, defined as the weighted sum of the total transient error and the variance of prediction error due to measurement noise errors, [18]. BBF is the constant g-h filter that satisfies:

(5) $$h=\frac{g^{2}}{2-g}$$

As *g *and *h *are related by Equation 5, the BBF has only one degree of freedom. CDF minimizes the least-squares fitting line of previous measurements, [16], giving old data lesser significance when forming the total error sum. This is achieved with weight factor *θ. *Parameters in the g-h filter are related by Equation 6. Selection of filter gain for the CDF is analogous to that for the BBF.

(6) $$\begin{array}{l} g=1-\theta^{2}\\ h={\left(1-\theta \right)}^{2} \end{array}$$

For the selection of the filter and for tuning of the correspondent parameters, the *Kinematic Estimation Error (KTE) *was used, (Equation 7). KTE quantifies the transient response through ${\bar{\left|\varepsilon \right|}}^{2}$ and, at the same time, the averaging of filtering capabilities of the filter through the term *σ*^2 ^[19].

(7) $$KTE=\sqrt{{\bar{\left|\varepsilon \right|}}^{2}+\sigma^{2}}$$

Where, $\bar{\left|\varepsilon \right|}$ is the mean square of errors of the filtered signal and *σ*^2 ^is the variance both related to a reference signal obtained through offline filtering the signal with the algorithm known as *zero-phase forward and reverse digital filtering*, [20]. This last filtering algorithm is non-causal once the signal is filtered both in forward and reverse directions in time and it can be only used in offline applications. Nevertheless, it offers an optimal reference signal for the proper selection of filter's coefficients, considering that the filter yields precisely zero-phase distortion.

The KTE was used for the selection of the filtering parameters for BBF and CDF: *g *and *θ *were modified within a broad range, using a small step, and the best solution was selected for each user, experiment and repetition. Once the best solution was found for each case, the delay between input and output signals was calculated. The selection of the best coefficients along with the KTE and delay (*δ*) for each user is presented in Table 1. Considering the components related to user's guidance intentions, the authors obtained empirically that the y-components of forces are the most important. Thus, for practical reasons, Table 1 only presents the mean values for the best filter coefficients, KTE and (*δ*) for the y-axis of each force sensor (right and left).

**Table 1:** Selection of best filter coefficients based on the KTE.

BBF				
**Subj**.	**Sensor**	**KTE [kgf]**	***δ*[ms]**	***g***

1	*F_Yright_*	(2.194 ± 0.1455)·10^-1^	(1.583 ± 0.1462)·10^1^	(2.817 ± 0.1795)·10^-2^

1	*F_Y left_*	(2.131 ± 0.0893)·10^-1^	(1.517 ± 0.1951)·10^1^	(2.900 ± 0.2828)·10^-2^

2	*F_Yright_*	(1.396 ± 0.1569)·10^-1^	(1.933 ± 0.3902)·10^1^	(2.517 ± 0.3727)·10^-2^

2	*FY _left_*	(1.333 ± 0.1414)·10^-1^	(1.850 ± 0.4113)·10^1^	(2.633 ± 0.4758)·10^-2^

3	*FY_right_*	(2.203 ± 0.4284)·10^-1^	(1.900 ± 0.4397)·10^1^	(2.425 ± 0.3521)·10^-2^

3	*FY _left_*	(2.291 ± 0.3513)·10^-1^	(1.933 ± 0.2134)·10^1^	(2.542 ± 0.2070)·10^-2^

4	*F_Yright_*	(2.105 ± 0.2947)·10^-1^	(1.967 ± 0.1972)·10^1^	(2.223 ± 0.1863)·10^-2^

4	*F_Y left_*	(2.019 ± 0.2605)·10^-1^	(1.967 ± 1.5986)·10^1^	(2.292 ± 0.1170)·10^-2^

5	*F_Yright_*	(2.183 ± 0.2209)·10^-1^	(2.383 ± 0.2544)·10^1^	(2.041 ± 0.1538)·10^-2^

5	*F_Yleft_*	(2.284 ± 0.2446)·10^-1^	(2.100 ± 0.3215)·10^1^	(2.229 ± 0.2634)·10^-2^

Mean values		(2.014 ± 0.4194)·10^-1^	(1.9133 ± 0.3721)·10^1^	(2.469 ± 0.3781)·10^-2^

**CDF**				

Subj.	Sensor	KTE [kgf]	*δ*[ms]	*θ*

1	*F_Yright_*	(2.124 ± 0.1447)·10^-1^	(2.050 ± 0.2432)·10^1^	(9.857 ± 0.01374)·10^-1^

1	*F_Y left_*	(2.056 ± 0.0896)·10^-1^	(1.967 ± 0.2494)·10^1^	(9.850 ± 0.01528)·10^-1^

2	*F_Yright_*	(1.321 ± 0.1437)·10^-1^	(2.533 ± 0.4988)·10^1^	(9.877 ± 0.02054)·10^-1^

2	*F_Y left_*	(1.262 ± 0.1311)·10^-1^	(2.217 ± 0.3891)·10^1^	(9.862 ± 0.02267)·10^-1^

3	*F_Yright_*	(2.108 ± 0.3627)·10^-1^	(2.533 ± 0.6600)·10^1^	(9.882 ± 0.02340)·10^-1^

3	*F_Y left_*	(2.174 ± 0.3218)·10^-1^	(2.617 ± 0.3184)·10^1^	(9.878 ± 0.01344)·10^-1^

4	*F_Yright_*	(2.035 ± 0.2687)·10^-1^	(2.567 ± 0.3815)·10^1^	(9.890 ± 0.01633)·10^-1^

4	*F_Y left_*	(1.947 ± 0.2533)·10^-1^	(2.433 ± 0.1795)·10^1^	(9.883 ± 0.00745)·10^-1^

5	*F_Yright_*	(2.094 ± 0.2037)·10^-1^	(2.967 ± 0.3543)·10^1^	(9.900 ± 0.01155)·10^-1^

5	*F_Y left_*	(2.177 ± 0.2495)·10^-1^	(2.783 ± 0.3288)·10^1^	(9.899 ± 0.01344)·10^-1^

Mean values		(1.923 ± 0.4002)·10^-1^	(2.467 ± 0.4847)·10^1^	(9.877 ± 0.0225)·10^-1^

Vilues (mean ± std. deviation) for KTE and delay obtained in the selection of g-h filters for high-frequency noise cancelation. As it can be seen, although the values of KTE are equivalent for both filtering algorithms, the delay is significantly smaller for the Benedict-Bordner Filter.

**Figure 4:**
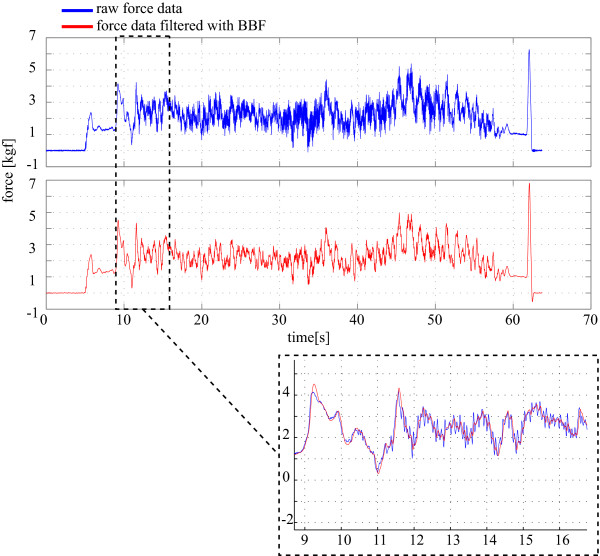
**Signal filtered with the selected g-h filter (Benedict-Bordner, *g *= *g *= 2.469·10^-2^)**. Dashed box shows a detail of the filtered signal (in red) compared with the original raw force data (in blue).

As it can be seen, KTE for BBF and CDF are very similar. Nevertheless, delay is significantly smaller when using the BBF approach. In addition, to avoid the need of tuning the algorithm for each user, sensor and repetition, the KTE and delay were recalculated using the mean values for the coefficients for each filter (*g *= 2.469·10^-2 ^for BBF and *θ *= 9.877·10^-1 ^for CDF). In this case, mean KTE of (2.035 ± 0.358)·10^-2^*kgf *and delay of (1.897 ± 0.3697)·10^1^*ms *were obtained for the BBF, while mean KTE of (1.951 ± 0.350)·10^-1^*kgf *and delay of (2.413 ± 0.131)·10^1^*ms *were obtained for the CDF. As the KTE in both cases are very similar and the delay is still significantly smaller for the BBF, this filter showed to be a better solution than CDF for the data obtained from the experiments performed in this work. Figure 4 shows an example of the data filtered with the selected filter.

#### Estimation of force component related to gait cadence

Once the filter for cancelation of high-frequency noise is introduced, this section presents a methodology for the estimation of the force component related with user's gait cadence. For that purpose, taking advantage of the periodicity of cadence, an adaptive filter based on the *Fourier Linear Combiner *(FLC) was applied. FLC is an adaptive algorithm used for continuous estimation of quasi-periodical signals based on a M harmonics dynamic Fourier model (Equation 8). Using frequency and number of harmonics as inputs for the model, the algorithm adapts amplitude and phase for each harmonic at the given frequency.

(8) $$s=\sum_{r=1}^{M}\left[w_{r}sin(r\omega_{0}k)\;+w_{M+1}cos(r\omega_{0}k)\right]$$

**Figure 5:**
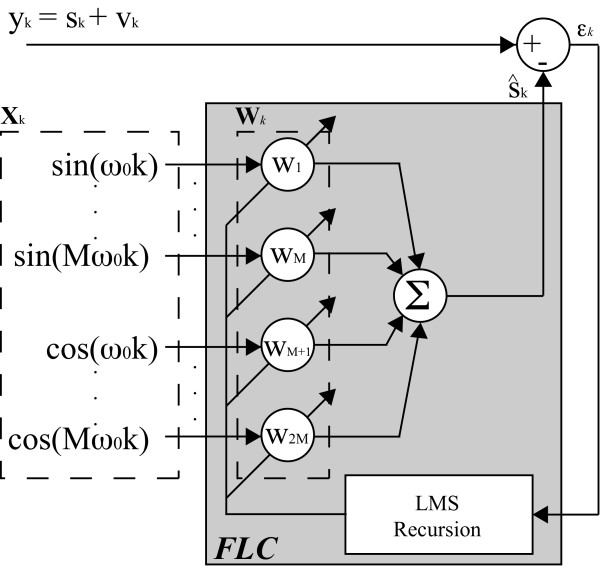
**The Fourier Linear Combiner algorithm**. The adaptation of the coefficients *w_k _*is performed based on the least-mean-square (LMS) recursion. *y_k _*is the input signal. The adaptive weight vector, **W***_k_*, generates a linear combination of the harmonic orthogonal sinusoidal components of the reference input vector, **X***_k_*. *M *is the number of the harmonics used and *μ *represents the amplitude adaptation gain used for the LMS recursion.

The adaptation of the coefficients *w_k _*is performed based on the least-mean-square (LMS) recursion, a descend method based on a special estimate of the gradient, [21], which ensures inherent zero phase. Figure 5 illustrates the FLC algorithm and shows how the LMS recursion is used for the adaptation of amplitude and phase. The equations for the FLC algorithm, presented in Figure 5 are described below.

(9) $$x_{rk}=\{\begin{array}{l} \text{sin }\left(r\omega_{0}k\right)\\ \text{cos}\left(\left(r-M\right)\omega_{0}k\right), \end{array}\begin{array}{r} 1\leq r\leq M\\ M+\;\leq r\leq 2M \end{array}$$

(10) $$\varepsilon_{k}=y_{k}-W_{k}^{\text{T}}X_{k}$$

(11) $$W_{k+1}=W_{k}+2\mu \varepsilon_{k}X_{k}$$

Where *y_k _*is the input signal. The adaptive weight vector, **W***_k_*, generates a linear combination of the harmonic orthogonal sinusoidal components of the reference input vector, **X***_k_*. As previously described, *M *is the number of the harmonics used and, finally, *μ *represents the amplitude adaptation gain used for the LMS recursion.

As mentioned before, the algorithm needs a frequency input for the correct estimation of the gait related force component. On the one hand, such information can be offered by an external system, such as a podometer (or any step counter). In this context, the authors proposed in [22] an ultrasonic subsystem that offer continuously the distance between each user's feet and the walker. From that information, cadence can be easily extracted and used for the FLC algorithm. The main disadvantage of this approach is that the user has to wear sensors on each feet compromising the usability of the device.

On the other hand, the author's also demonstrated in [13] that the vertical components of the force signals can be used for continuous estimation of gait cadence using the Weighted-Frequency Fourier Linear Combiner (WFLC). The WFLC is an extension of the FLC noise canceler presented before and also tracks frequency of the input signal based on a LMS recursion. Therefore, the WFLC adapts in real-time its amplitude, frequency and phase, [23].

As the WLFC is designed to adapt to the dominant-frequency component in a signal [24], it is important to perform a previous stage of band-pass filtering (compatible with gait cadence frequencies) for the correct performance of the WFLC. Although this filtering stage can cause undesirable time delay in the force signals, instantaneous temporal changes in gait cadence (WFLC's frequency output) are minimal.

Therefore, an external branch of cadence estimation based force measurement, and the WFLC algorithm showed to be very useful in the application presented in this paper.

**Figure 6:**
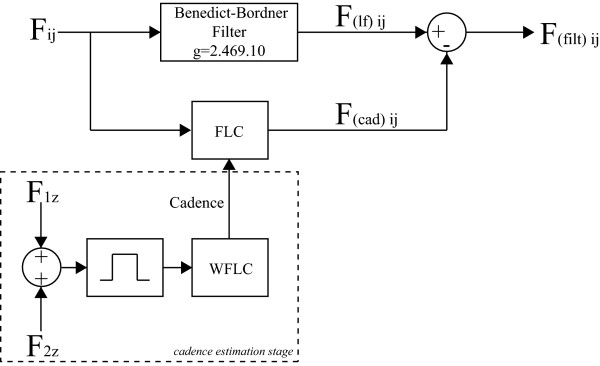
**Signal processing architecture for estimation of user's intent force component**. *F_ij _*is the force signal obtained from axis (j), sensor (i). $F_{{\left(\iota f\right)}_{ij}}$ denotes the force signal obtained after the high frequency noise cancelation and $F_{{\left(cad\right)}_{ij}}$ is the periodical, cadence-dependent component of force. Cadence estimation stage by means of WLFC is indicated with dashed lines.

Thus, the combination of WLFC and FLC presents great advantages, [25]. The band-pass filtering allows the WFLC to robustly adapt to the values of gait cadence, while the FLC operates on the raw input, ensuring zero-phase amplitude estimation, presented in Figure 3. Figure 6 shows the complete diagram of the filtering architecture used for the extraction of user's intentions from force data.

During the preliminary experiments, the method for filtering the force sensor data was observed to be very effective for canceling the cadence-related components in symmetrical gait, in which the user applies approximately the same amount of body weight to both supporting platforms. Nevertheless, when planning the filter design to help people with pathological gait, it is interesting to include the possibility for the cancelation of components related to asymmetrical supporting forces. When such situation occurs, it was experimentally observed that the oscillatory component presents more influence due to the cadence of one foot.

**Figure 7:**
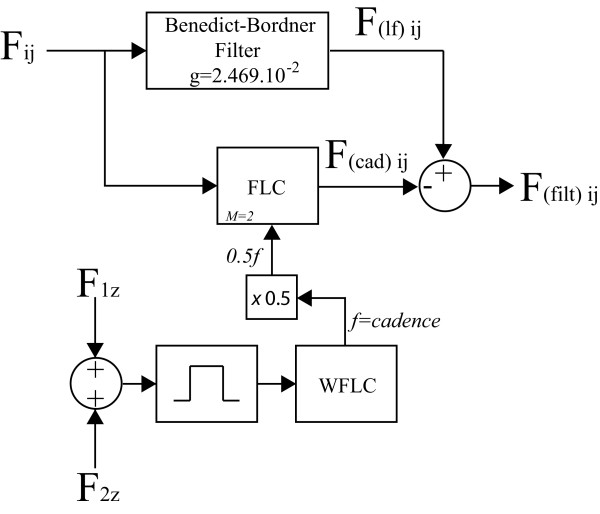
**Final signal processing architecture for estimation of user's intent force component considering the possibility of asymmetrical supporting forces**.

However, the presence of different gait cycle durations for left and right feet affects equally the number of steps per minute of each foot. In this manner, for the cancellation of asymmetrical supporting forces it is necessary to eliminate two frequency harmonics (*M *= 2): *f*_1 _and *f*_2 _equal to the half of cadence and cadence, respectively. The FLC algorithm cancels each component individually if they are within the force signals. Figure 7 presents the final diagram for cancelation of all undesired components of force data and taking into account the possibility of asymmetrical supporting forces.

In previous works, the authors presented a methodology for tuning the WFLC parameters for the online estimation of cadence from force data, [13]. This methodology consists in adjusting five parameters. Three of them do not require tuning: the number of harmonics of the model, M, which is fixed to 1, the instantaneous frequency at initialization, which is automatically set as the lower cut-off frequency of the band-pass filter (0.5 Hz in this work) and the bias weight to compensate for low frequency drifts, which is set to zero in this application. Finally, the amplitude and frequency update weights are adjusted in a manner that the frequency output of the WFLC adapts as fast as possible to the dominant-frequency component of the input signals.

Since the WFLC tuning was previously solved, authors start from the supposition that continuous cadence is a known parameter. Only FLC tuning is required. As the number of the harmonics (*M*) of the FLC is set to 2, only one parameter needs to be adjusted: the amplitude adaptation gain, *μ*, used for the LMS recursion. The selection of values for *μ *affects directly the convergency time and, most importantly, the bandwidth (*BW*) of the FLC adaptive filter, [26]. For values of *μ *≪1, the bandwidth is given by *BW *≈ 2*μ*.

Considering that, small values of *μ *imply in a narrow band filter and the cancelation of a very specific frequency. Nevertheless, if the force component signal is not a perfect sinusoidal wave, the cancelation will not be effective. Opposite to the g-h filters selection, in this case, no reference signal can be obtained for the automatic/optimal selection of the FLC parameter. As in other similar works, such as [19], the selection of *μ *is usually performed empirically.

**Figure 8:**
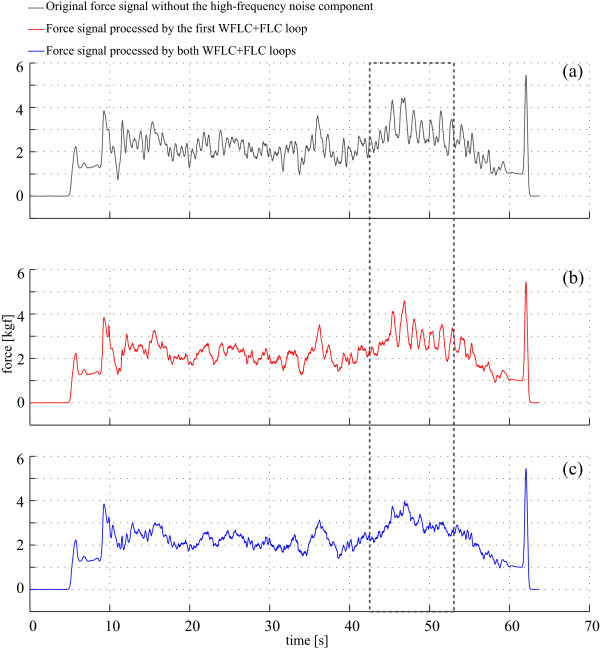
**The top graph (in black) shows the force data after the canceling of the high-frequency components for the tuning of the FLC algorithm**. Middle graph shows the signal (in red) after the FLC filtering taking into account only the cadence frequency (one harmonic). Finally, the bottom graph (in blue) shows the filter output signal processed with the final FLC filter. The dashed box indicates the instants in which asymmetrical support is forced through a right turn performed with the walker.

For the signals obtained in this study, *μ *= 0.002 presented excellent results for both FLC filters. Figure 8 shows the signal processed with the FLC algorithm. First, Figure 8(a) shows the original signal after the high frequency noise cancelation and, thus, the input of the first FLC filter. Second, Figure 8(b) shows the output of the FLC filter in which only the cadence component was canceled (scheme presented in Figure 6). As previously mentioned, in the experiments performed for this work a right/left turn was executed. This was done in order to intentionally generate different gait cycle duration for each foot and asymmetric supporting forces in the walker's handles. This turn is marked with the dotted line. As it can be seen, a slower oscillatory component is observed inside this dotted line in both Figure 8(a) and 8(b). The final FLC filtering architecture (Figure 7) tracks and cancels both harmonics of the cadence frequency ($f=\frac{cadence}{2}$ and *f *= *cadence*). The signal resulting from such filtering algorithm is shown in Figure 8(c).

## Results and Discussion

Once both filters were individually tuned, a methodology for the analysis of the complete filtering architecture is presented in this section. For that purpose, an index (*R *in Equation 12) based on the ratio of the energy within the cadence components of gait before and after the filters in the frequency-domain is proposed. *R *indicates the reduction of signal's energy within the cancelation range given by the frequency input of the FLC algorithm presented in this work.

(12) $$R=1-\frac{\sum_{\lambda =f_{1,1}}^{f_{1,2}}|X_{filt}\left[\lambda \right]|+\sum_{\lambda =f_{2,1}}^{f_{2,2}}|X_{filt}\left[\lambda \right]|}{\sum_{\lambda =f_{1,1}}^{f_{1,2}}|X\left[\lambda \right]|+\sum_{\lambda =f_{2,1}}^{f_{2,2}}\left|X\left[\lambda \right]\right|}$$

**Figure 9:**
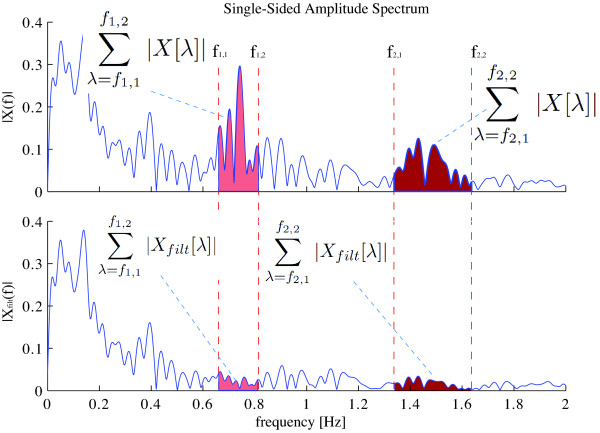
**Graphical representation of the methodology for quantifying the canceling of the cadence related components of force data**. *R *indicates the reduction of signal's energy within the cancelation range given by the frequency input of the FLC algorithm presented in this work.

*f*_1,1 _and *f*_1,2 _are the minimum and maximum values of cadence for the first harmonic of the FLC filter and *f*_2,1 _and *f*_2,2 _for the second harmonic (*f = cadence*). This method for the evaluation of the filtering strategy is presented graphically in Figure 9. The mean and standard deviation values of *R *obtained for the five subjects considering all the repetitions and right and left sensors are presented in Table 2. As it can be seen, a reduction close to 80% of the energy in the frequency components is obtained for all the subjects. In addition, small standard deviations indicate the robustness of the proposed methodology for the canceling of non-desired components from force data.

**Table 2:** Attenuation of cadence related components by means of WFLC-FLC algorithms.

Subject	R (mean ± std. deviation)
1	0.8084 ± 0.0140

2	0.7605 ± 0.1104

3	0.7915 ± 0.0147

4	0.8045 ± 0.0173

5	0.7970 ± 0.0204

Values (mean ± std. deviation) for ratio index *R *for each subject including all six repetitions of both experiments.

**Figure 10:**
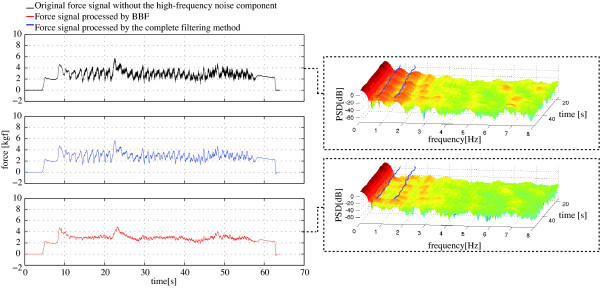
**On the left, representation of force data for the *y *axis of the left sensor in one experiment in three stages: raw force data (top), data after the BBF (middle) and final output (bottom)**. On the right, the spectrogram of the filtering input (top) and output (bottom) are presented along with the continuous line marking the continuous cadence estimation by means of WFLC. Important reductions in PSD of high frequencies and in both cadence harmonics are observed.

Finally, Figure 10 presents the effect of the complete filtering methodology over the acquired force signals. First, on the top left the raw force signals are presented. Along with that, the force signal after the processing by the BBF and the final filtered data are both presented. On the right side, the spectrogram, [20], of the input and output signals are presented. In these graphs, the continuous cadence output of both harmonics obtained by means of WFLC are marked with the continuous lines. As it can be seen in the top spectrogram, these signals coincide with two zones of local maxima. In the bottom spectrogram, the attenuation of both cadence component by means of the FLC algorithm is observed. In addition, the reduction in PSD is also noticed in the higher frequencies due to the cancelation of the vibration components through the Benedict-Bordner Filter.

## Conclusions

**Figure 11:**
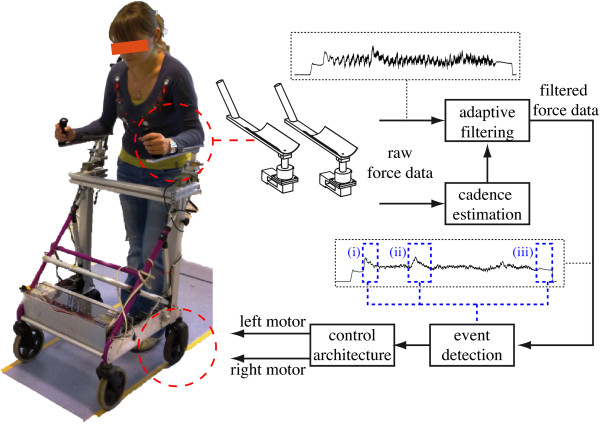
**Complete system architecture implemented in the SIMBIOSIS walker's firmware**. The output signals of the filtering architecture presented in this paper is used by a event detection algorithm that classify the signals in real-time and send navigational commands to the controller that commands directly the walker's dc motors. For illustration purposes, three areas are distinguish with blue boxes: (i) start walking, (ii) right turn and (iii) stop walking.

For achieving safer and more reliable robotic walkers for disabled people, it is important to develop efficient methods for inferring of user's voluntary commands from user-walker interaction in assisted gait. Regarding usability issues and in order to extend the use of smart walkers outside clinics and research laboratories, such information must be ideally extracted without installing any sensors in user's body. In this context, this paper presents a novel strategy for the estimation of the voluntary component of force data based on two filtering stages. First, the higher frequency signals due to walker vibrations caused by floor imperfections are cancelled by means of a Benedict-Bordner g-h filter. Second, a WFLC-FLC algorithm is built for the estimation of cadence related force components. Such components present interesting information on spatio-temporal parameters of user's gait and they have been previously studied and characterized by the authors in previous works. In this work, as the objective is to infer voluntary guidance information, those cadence related components have been subtracted from the force data. All the filtering strategies presented in this work are currently working in real-time programmed into the firmware installed on the Simbiosis walker. The first experiments of controlled motion of the device are being performed with healthy subjects. Figure 11 shows an example of the complete architecture implemented in the SIMBIOSIS walker's firmware. As it can be seen, the force data processed with the algorithms presented in this work will be used as input signals for an event detection algorithm that classify, in real-time, the navigational commands applied by the user during gait. Notice that is impossible to extract such information from the raw data. In Figure 11, it is shown an example of three areas in which navigational transition events occur: (i) start walking, (ii) right turn and (iii) stop walking. Once this validation is over, the device will be taken to a clinical environment for the final validation with user's in which gait patterns, weight bearing and stability will be evaluated in collaboration with the medical staff involved in the research project.

## Competing interests

The authors declare that they have no competing interests.

## Authors' contributions

AF designed the study, performed the experiments and was responsible for the data analysis and calculations. JAG and ER contributed to data analysis and along with AF drafted the manuscript. JLP and RC contributed to the discussion and the interpretation of the results. AF, JAG and ER shared the discussion. All authors read and approved the final manuscript.
